# Two-Year Clinical Follow-Up Assessment of the Novel Cingular Surgical Bovine Pericardial Valve

**DOI:** 10.3389/fcvm.2021.736877

**Published:** 2021-12-13

**Authors:** Jinmiao Chen, Minzhi Lv, Yuntao Lu, Jiahui Fu, Yingqiang Guo, Liang Tao, Xinmin Zhou, Tianxiang Gu, Lai Wei, Tao Hong, Chunsheng Wang

**Affiliations:** ^1^Department of Cardiovascular Surgery, Zhongshan Hospital, Fudan University, Shanghai, China; ^2^Department of Biostatistics, Zhongshan Hospital, Fudan University, Shanghai, China; ^3^Department of Cardiac Surgery, West China Hospital of Sichuan University, Chengdu, China; ^4^Department of Cardiac Surgery, Wuhan Asia Heart Hospital, Wuhan, China; ^5^Department of Cardiac Surgery, The Second Xiangya Hospital of Central South University, Changsha, China; ^6^Department of Cardiac Surgery, The First Hospital of China Medical University, Changchun, China

**Keywords:** bovine pericardial valve, surgical valve replacement, safety, effectiveness, hemodynamic performance

## Abstract

**Objectives:** To evaluate the 2-year clinical safety and hemodynamic outcomes of the Cingular bovine pericardial bioprosthesis.

**Methods:** A prospective, multicenter, single-arm trial was conducted in patients who required aortic or mitral valve replacement. From March 2016 to October 2017, 197 patients were implanted with the Cingular bovine pericardial valve at five sites in China. The clinical outcomes and hemodynamic performance were assessed through a 2-year follow-up. Clinical safety events were reviewed by an independent clinical events committee, and echocardiographic data were assessed by an independent core laboratory.

**Results:** The mean age was 66.9 ± 4.9 years. The 2-year survival rate was 96.4%. A complete 2-year clinical follow-up was achieved in 189 of 190 survivors. No case of structural valve deterioration, major perivalvular leak, prosthetic valve endocarditis, or valve-related reoperation was seen. For the aortic valve, the mean pressure gradient observed was 12.5 ± 4.0 mm Hg, and the effective orifice area (EOA) was 2.0 ± 0.3 cm^2^. For the smaller size aortic valves, 19 mm and 21 mm, respective mean EOA values of 1.7 ± 0.2 cm^2^ and 1.8 ± 0.2 cm^2^ were found. The values for mean pressure gradient and mean EOA for mitral bioprostheses were 4.0 ± 1.4 mm Hg and 2.2 ± 0.3 cm^2^, respectively. There was no significant change between 1-year and 2-year hemodynamic performance.

**Conclusions:** The Cingular bovine pericardial valve showed favorable clinical safety and hemodynamic outcomes over a 2-year follow-up. Further follow-up is required to validate the long-term durability.

## Introduction

Even though the emergence of transcatheter valve replacement, surgical valve replacement is still a well-established treatment for diseased heart valve. Compared with mechanical valves, the bioprosthetic heart valve has the advantage of no lifetime anticoagulation treatment. Durability and hemodynamic performance are the major determinants of a good bioprosthetic heart valve (1–4). However, an ideal bioprosthetic heart valve for replacement of the native heart valve remains elusive. Thus, continuous modifications and improvements are advocated (5–7). Due to the aging population and emerging valve-in-valve technique, the demand for bovine pericardial valves is expected to increase (8). However, few surgical bovine pericardial valves have been developed in China, and the prospective clinical trial outcome for these valves is lacking (9).

In reference to the Carpentier-Edwards Perimount (Edwards Lifesciences Corporation, Irvine, USA) valve platform, the Cingular bovine pericardial valve (Shanghai Cingular Biotech Corporation, Shanghai, China) was specifically designed to offer a number of alterations to enhance the stability and the durability of the valve, mitigate the potential for an occurrence of triangular leaflet opening, and thereby promote enlargement of the effective orifice area (EOA) (10). Prior to applying the valve clinically in humans, it had been successfully evaluated in the juvenile sheep model (11). A clinical study was subsequently conducted to evaluate the safety and effectiveness of this novel valve design. An early report from this trial demonstrated a good safety profile and hemodynamic performance of the study valve (11). The current report further details the 2-year clinical and hemodynamic outcome of the Cingular bovine pericardial valve.

## Methods and Materials

### Study Design and Population

**Figure 1:**
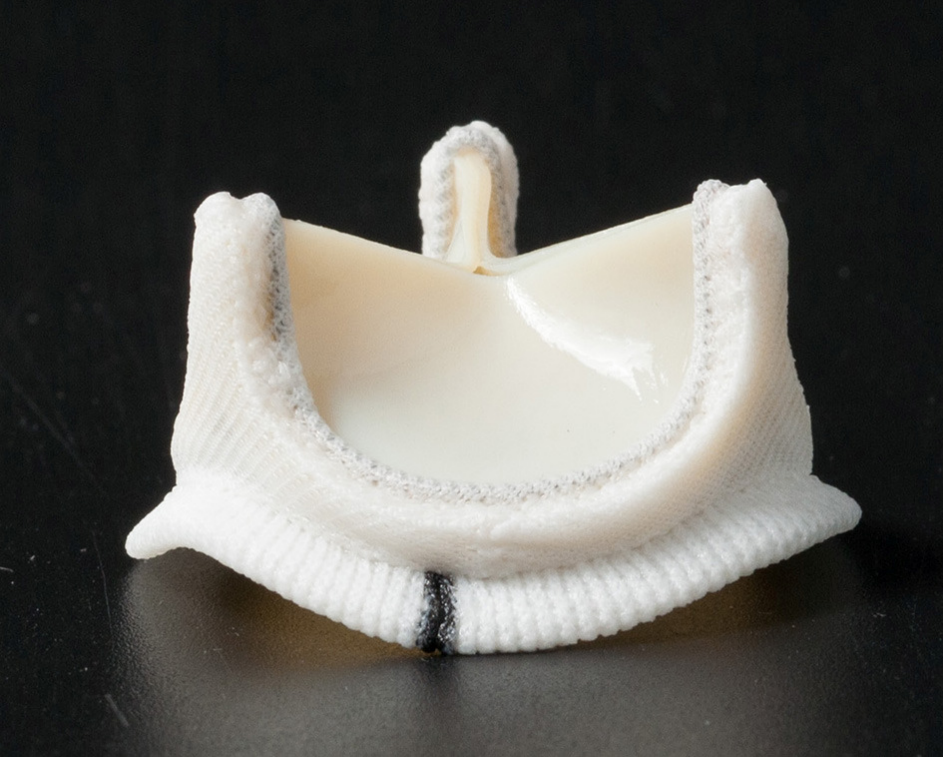
The Cingular bovine pericardial valve.

This clinical study (Clinical Trial No.: NCT02755220) has been set up as a prospective, multicenter, single-arm clinical investigation to assess the safety and hemodynamic performance of the Cingular bovine pericardial valve (Figure 1). In accordance with Good Clinical Practice guidelines, the study protocol was submitted to an Institutional Review Board or Ethics Committee at each investigational site. Subsequent approval was obtained for all study sites. The patients were enrolled in the study conditional upon having given their informed consent in writing prior to surgery and based on the clinical justification for surgical valve replacement. The inclusion criteria and the exclusion criteria were described previously (11).

### Study Device and Surgical Procedure

In contrast to the Carpentier-Edwards Perimount valve, the Cingular bovine pericardial valve incorporated new optimizations, including the design of a three-layer stent structure, improved leaflet matching, and redesign of the sewing ring (10). These modifications made the EOA increase by about 25% over the Carpentier-Edwards Perimount valve of the same size during *in vitro* tests. Furthermore, a novel three-layer stent structure, in which the alloy wire was fixed by both the internal and external polyester rings, made the whole structure more stable (10).

Aortic or mitral valve replacement was performed at the discretion of the investigator using either routine median sternotomy or upper hemisternotomy. Ascending aortic and venous cannulation was performed, standard cardiopulmonary bypass commenced, and mild hypothermia applied, followed by an induction of cardiac arrest by antegrade or combined with retrograde infusion of del Nido or Buckberg blood cardioplegia. All surgical procedures were performed at five large cardiac surgery centers in China. The appropriate bioprosthesis size was carefully chosen for each individual patient using the specifically designed sizers. Interrupted, pledgeted, mattressed sutures were used in all valve implants. In accordance with the Guidelines for Management of Patients with Valvular Heart Disease, anticoagulation for 3 months postoperatively was recommended (12, 13).

### Safety and Hemodynamic Endpoints

During follow-up, the following safety endpoints were evaluated: all-cause mortality, structural valve deterioration (SVD), major perivalvular leak, prosthetic valve endocarditis, major hemorrhage event, thromboembolic event, valve thrombosis, and valve-related reoperation. Evaluation of the safety endpoints was performed in accordance with the reporting guidelines for mortality and morbidity after valve replacement surgery and the updated objective performance criteria for clinical evaluation of novel heart valve prostheses (14, 15). The definition of SVD included dysfunction or deterioration involving the operated valve (exclusive of infection or thrombosis), as determined by reoperation, autopsy, or clinical investigation (14).

Patient follow-up was established by postoperative transthoracic Doppler echocardiography at 1 month, 1 year, and 2 years. Evaluation of the data was performed by an independent Echocardiographic Core Laboratory (Department of Echocardiography, Zhongshan Hospital, Fudan University). The hemodynamic performance was assessed by the following parameters: mean pressure gradient, EOA, peak flow velocity, and pressure half time. The mean pressure gradient across the bioprosthesis was obtained using the modified Bernoulli equation.

### Data Management and Statistical Analysis

Monitoring of the study was done in accordance with Good Clinical Practice by an independent Clinical Research Organization. Data collection in each of the participating study site was done by the respective study coordinator. An independent Clinical Events Committee adjudicated all suspected endpoint events in the study using the specific hospital clinical file source documents of each case. Data management and statistical analyses were performed by the National Center for Cardiovascular Diseases (Beijing, China).

Continuous variables were summarized as mean ± standard deviation and median (Q1,Q3). Categorical variables were reported as the number and percentage of subjects in each category. Kaplan–Meier analysis was applied to estimate the survival rate after valve implantation. The Mann–Kendall trend test was used to test the increasing or decreasing trend of the hemodynamic performance after valve implantation. Two-sided *p*-values < 0.05 were considered to represent statistical significance. Tests were performed using SAS 9.4 (SAS Institute, Cary, North Carolina). R software, version 3.6.1 (R Foundation for Statistical Computing, Vienna, Austria).

## Results

### Patient Characteristics at the Baseline

Between March 2016 and October 2017, a total of 197 patients requiring surgical aortic or mitral valve replacement received the Cingular bovine pericardial prosthesis. At the implantation, the mean age of the patients was 66.9 ± 4.9 years, ranging from 60 to 84 years; 40.6% of the patients were females. NYHA functional class II, III, and IV at the baseline were 25.9, 73.1, and 1.0%, respectively. For the study cohort, the mean preoperative Society of Thoracic Surgeons Predicted Risk of Mortality (STS-PROM) score was 1.6 ± 1.1%, while the mean Logistic European System for Cardiac Operative Risk Evaluation (EuroSCORE) II was 2.8 ± 2.3%. The preoperative patient characteristics are shown in Table 1.

**Table 1:** Baseline characteristics of all patients (*N* = 197).

**Parameter**	**All (*N* = 197)**	**AVR (*N* = 148)**	**MVR (*N* = 36)**	**DVR (*N* = 13)**
Age, years	66.9 ± 4.9	67.7 ± 5.1	65.2 ± 3.7	63.3 ± 2.3
Median (Q1,Q3)	66.0 (63.1,69.8)	66.8 (64.0,70.8)	64.3 (62.6,67.2)	62.6 (61.7,65.5)
Female	80 (40.6%)	54 (36.5%)	18 (50%)	8 (61.5%)
BMI, kg/m^2^	23.1 ± 3.5	23.2 ± 3.7	23.3 ± 3.0	22.0 ± 2.8
Median (Q1,Q3)	23.0 (20.8,25.1)	23.0 (20.6,27.6)	23.0 (21.8,24.4)	22.5 (20.8,23.6)
**NYHA functional class**				
II	51 (25.9%)	36 (24.3%)	13 (36.1%)	2 (15.4%)
III	144 (73.1%)	111 (75.0%)	22 (61.1%)	11 (84.6%)
IV	2 (1.0%)	1 (0.7%)	1 (2.8%)	0 (0%)
Systemic hypertension	74 (37.6%)	62 (41.9%)	11 (30.6%)	1 (7.7%)
Coronary artery disease	8 (4.1%)	7 (4.7%)	1 (2.8%)	0(0%)
COPD	29 (14.7%)	26 (17.6%)	2 (5.6%)	1 (7.7%)
Diabetes mellitus	9 (4.6%)	6 (4.1%)	2 (5.6%)	1 (7.7%)
Logistic EuroSCORE II	2.8 ± 2.3	3.0 ± 2.5	2.5 ± 1.5	2.2 ± 1.2
Median(Q1,Q3)	2.0 (1.5,3.2)	1.9 (1.5,3.3)	2.1 (1.4,3.2)	2.1 (1.4,2.6)

*BMI, body mass index; NYHA, New York Heart Association; COPD, chronic obstructive pulmonary disease; EuroSCORE, European System for Cardiac Operative Risk Evaluation*.

### Procedural Outcomes

Valve implantation surgery was successful in 100% of the patients. In 75.1% (148/197) of the patients, aortic valve replacement was performed, while mitral valve and double valve replacement represented 18.3% (36/197) and 6.6% (13/197) of the patients, respectively. A full sternotomy was performed in 98.5% of cases and an upper hemisternotomy in 1.5% of cases. In total, 161 aortic bioprostheses were implanted in the aortic position (19-25 mm) and 49 in the mitral position (25-29 mm). For the aortic prosthesis group, 37.8% of the patients received either a 19-mm or a 21-mm size prosthesis. In addition, the following concomitant procedures were performed: Tricuspid valve repair (*n* = 54, 27.4%), atrial fibrillation ablation (*n* = 32, 16.2%), mitral valve repair (*n* = 22, 11.2%), ascending aortoplasty (*n* = 16, 8.1%), Bentall procedure (*n* = 16, 8.1%), left ventricular outflow tract myectomy (*n* = 2, 1.0%), atrial septal defect repair (*n* = 1, 0.5%) or ventricular septal defect repair (*n* = 1, 0.5%). For the overall group of 197 patients, the mean aortic cross clamp time and cardiopulmonary bypass time were 70.2 ± 26.2 and 102.8 ± 30.9 min, respectively (Table 2).

**Table 2:** Intraoperative data (*N* = 197).

**Parameter**	**Result**
CPB time, min	102.8 ± 30.9
Median(Q1,Q3)	98.0 (78.0,124.0)
Crossclamp time, min	70.2 ± 26.2
Median(Q1,Q3)	65.0 (51.0,87.0)
**Operations**	
AVR	148 (75.1%)
MVR	36 (18.3%)
DVR	13 (6.6%)
Aortic valve sizes	161
19A, mm	21 (13.0%)
21A, mm	40 (24.8%)
23A, mm	66 (41.0%)
25A, mm	34 (21.1%)
Mitral valve sizes	49
25M, mm	12 (24.5%)
27M, mm	34 (69.4%)
29M, mm	3 (6.1%)

*CPB, cardiopulmonary bypass; AVR, aortic valve replacement; MVR, mitral valve replacement; DVR, double valve (aortic and mitral valve) replacement; A, aortic valve; M, mitral valve*.

### Safety Outcomes

**Figure 2:**
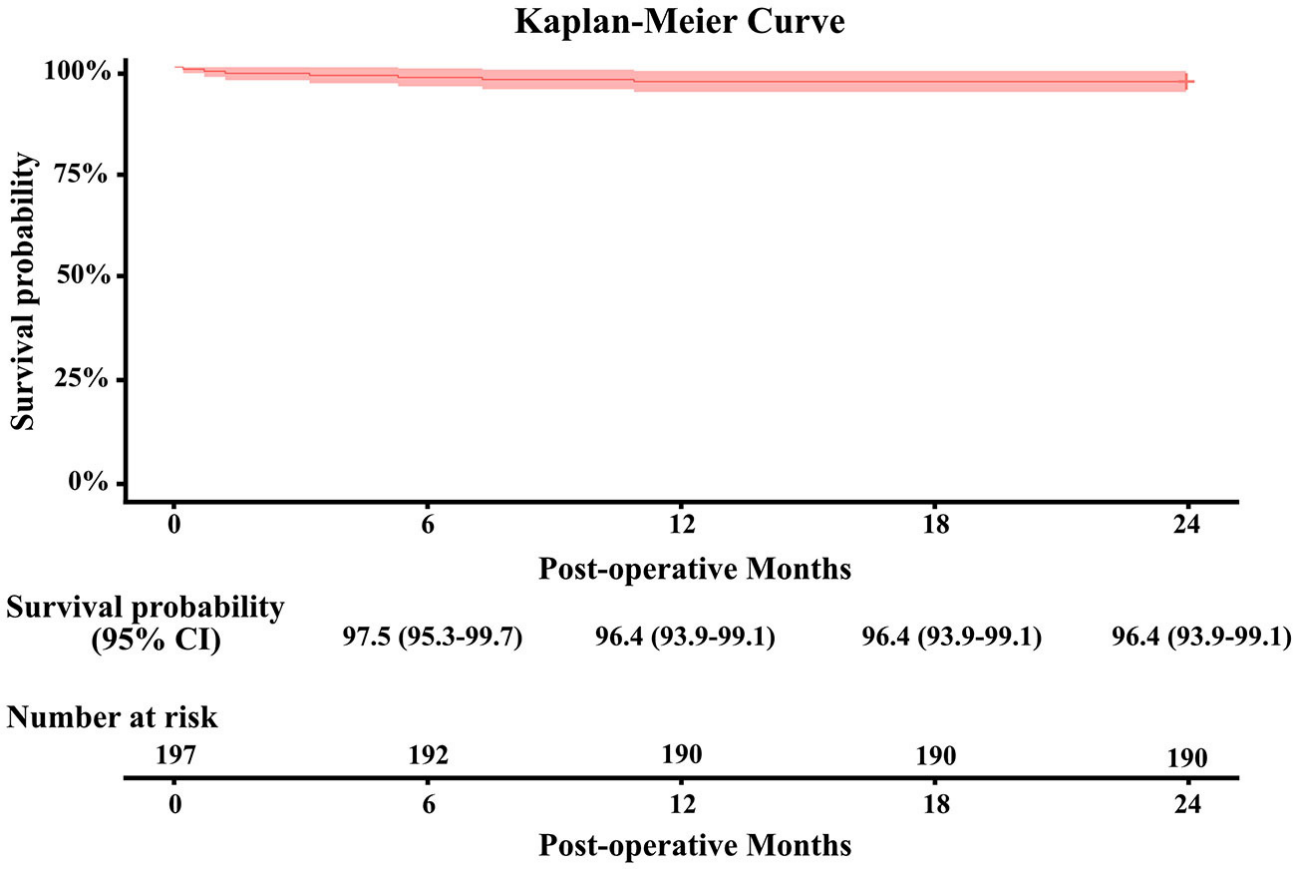
Kaplan–Meier survival curve ± fitted 95% confidence interval for the Cingular bovine pericardial valve.

The 2-year all-cause mortality rate was 3.6% (7/197) (Figure 2). Patient death occurred in one patient due to heart failure, one due to malignant arrhythmia, one due to aortic dissection, one due to sepsis, one due to stroke, and two due to unknown reasons. Overall, 99.5% (189/190) of the patients completed 2-year clinical follow-up after valve implantation. There was no case of structural valve deterioration, major perivalvular leak, or prosthetic valve endocarditis. Also, no thromboembolic event, major hemorrhage, or valve-related reoperation was reported. The incidence of valve thrombosis was 0.5% (1/197) in one patient requiring MVR with postoperative atrial fibrillation.

### Hemodynamic Outcomes

The echocardiographic data for all the subjects presented by valve position and size are shown in Tables 3, 4. A complete 2-year echocardiographic follow-up was achieved in 184 of 190 survivors (96.8%). The mean gradient and EOA for aortic prostheses at 2 years were 12.5 ± 4.0 mm Hg and 2.0 ± 0.3 cm^2^, respectively. The mean EOA for 19- and 21 mm sizes of aortic prostheses at 2 years was 1.7 ± 0.2 cm^2^ and 1.8 ± 0.2 cm^2^, respectively. For the mitral valves, the hemodynamic performance was also favorable with a mean gradient and EOA at 2 years of 4.0 ± 1.4 mm Hg and 2.2 ± 0.3 cm^2^, respectively. After implantation, there were no statistically significant differences in EOA or mean gradient across all valve sizes at 1-month, 1-year, and 2-year follow-up.

**Table 3:** Hemodynamic performance in aortic valve position.

**Time**	**Parameters**	**All sizes**	**19 mm**	**21 mm**	**23 mm**	**25 mm**
1-month	PFV, cm/s	2.3 ± 0.4 (*n* = 157)	2.7 ± 0.3 (*n* = 20)	2.4 ± 0.4 (*n* = 40)	2.3 ± 0.3 (*n* = 64)	2.1 ± 0.3 (*n* = 33)
	Median (Q1,Q3)	2.3 (2.1,2.6)	2.7 (2.5,2.9)	2.4 (2.1,2.7)	2.3 (2.1,2.5)	2.1 (2.0,2.3)
	MG, mm Hg	11.6 ± 3.5	15.2 ± 3.4	12.1 ± 3.7	11.2 ± 2.8	9.3 ± 2.8
	Median (Q1,Q3)	11.0 (9.0,14.0)	15.7(12.7,17.5)	11.6(9.2,15.0)	11.0(9.3,13.2)	9.2(7.5,10.2)
	EOA, cm^2^	2.0 ± 0.3	1.7 ± 0.2	1.8 ± 0.1	2.1 ± 0.2	2.3 ± 0.2
	Median (Q1,Q3)	2.0(1.8,2.2)	1.7(1.5,1.8)	1.8(1.7,1.9)	2.1(1.9,2.2)	2.3(2.2,2.4)
1-year	PFV, cm/s	2.5 ± 0.4 (*n* = 151)	2.8 ± 0.4 (*n* = 19)	2.5 ± 0.4 (*n* = 39)	2.4 ± 0.3 (*n* = 61)	2.2 ± 0.3 (*n* = 32)
	Median (Q1,Q3)	2.4(2.2,2.7)	2.8(2.6,3.1)	2.6(2.1,2.8)	2.4(2.2,2.6)	2.2(2.1,2.4)
	MG, mm Hg	12.8 ± 4.4	17.0 ± 3.8	13.1 ± 4.0	12.7 ± 4.4	10.0 ± 2.9
	Median (Q1,Q3)	11.8(9.9,15.0)	17.0(13.7,20.0)	13.0(10.0,15.1)	11.6(10.0,14.0)	9.9(7.8,11.4)
	EOA, cm^2^	1.9 ± 0.3	1.6 ± 0.2	1.8 ± 0.1	1.9 ± 0.2	2.2 ± 0.2
	Median (Q1,Q3)	1.9(1.7,2.1)	1.6(1.5,1.7)	1.8(1.7,1.9)	1.9(1.8,2.0)	2.3(2.1,2.4)
2-year	PFV, cm/s	2.4 ± 0.4 (*n* = 148)	2.7 ± 0.4 (*n* = 18)	2.4 ± 0.4 (*n* = 39)	2.4 ± 0.3 (*n* = 60)	2.2 ± 0.3 (*n* = 31)
	Median (Q1,Q3)	2.4(2.1,2.7)	2.6(2.5,3.0)	2.5(2.2,2.7)	2.4(2.2,2.7)	2.0(1.9,2.1)
	MG, mm Hg	12.5 ± 4.0	15.2 ± 4.5	13.0 ± 4.1	12.6 ± 3.5	10.1 ± 3.4
	Median(Q1,Q3)	12.0(9.2,15.0)	14.6(12.0,19.0)	12.7(10.0,15.0)	12.0(10.5,14.9)	8.5(7.6,12.9)
	EOA, cm^2^	2.0 ± 0.3	1.7 ± 0.2	1.8 ± 0.2	2.0 ± 0.2	2.3 ± 0.2
	Median(Q1,Q3)	2.0(1.7,2.1)	1.6(1.5,1.8)	1.8(1.6,1.9)	2.0(1.8,2.1)	2.3(2.1,2.4)
*P*-value for trend^*^	PFV	1.000	1.000	1.000	0.540	0.540
	MG	1.000	1.000	1.000	1.000	0.296
	EOA	1.000	1.000	1.000	1.000	1.000

*PFV, peak flow velocity; MG, mean gradient; EOA, effective orifice area*.

* *Mann–Kendall test for trend analysis*.

**Table 4:** Hemodynamic performance in mitral valve position.

**Time**	**Parameters**	**All sizes**	**25 mm**	**27 mm**	**29 mm**
1-month	PHT, ms	105.2 ± 23.4 (*n* = 48)	105.7 ± 19.6 (*n* = 12)	103.7 ± 25.0 (*n* = 33)	119.7 ± 20.6 (*n* = 3)
	Median(Q1,Q3)	108.0(91.3,118.0)	109.0(89.4,118.0)	100.0(90.9,115.0)	130.0(96.0,133.0)
	MG, mm Hg	4.2 ± 1.3	3.8 ± 1.5	4.4 ± 1.3	3.7 ± 0.4
	Median(Q1,Q3)	3.9(3.3,4.9)	3.6(3.1,4.1)	4.0(3.5,5.3)	3.6(3.3,4.1)
	EOA, cm^2^	2.3 ± 0.4	2.2 ± 0.3	2.4 ± 0.4	2.3 ± 0.2
	Median(Q1,Q3)	2.4(2.1,2.5)	2.2(2.0,2.6)	2.4(2.2,2.5)	2.4(2.1,2.4)
1-year	PHT, ms	111.0 ± 24.4 (*n* = 49)	112.1 ± 16.5 (*n* = 12)	108.6 ± 25.4 (*n* = 34)	133.0 ± 35.5 (*n* = 3)
	Median(Q1,Q3)	108.0(94.0,124.0)	111.5(101.0,122.0)	106.0(91.0,123.0)	153.0(92.0,154.0)
	MG, mm Hg	4.3 ± 2.0	4.3 ± 1.6	4.4 ± 2.2	3.2 ± 0.6
	Median(Q1,Q3)	3.9(3.3,4.7)	4.1(3.6,4.8)	4.0(3.3,4.7)	3.2(2.7,3.8)
	EOA, cm^2^	2.1 ± 0.3	2.1 ± 0.4	2.1 ± 0.3	2.1 ± 0.4
	Median(Q1,Q3)	2.2(1.8,2.4)	2.2(1.8,2.5)	2.2(1.9,2.4)	2.1(1.7,2.4)
2-year	PHT, ms	116.1 ± 28.1 (*n* = 48)	112.3 ± 19.0 (*n* = 12)	116.7 ± 29.2 (*n* = 33)	125.7 ± 52.5 (*n* = 3)
	Median(Q1,Q3)	111.0(94.6,133.3)	108.3(98.9,123.5)	111.0(93.2,134.5)	126.0(73.0,178.0)
	MG, mm Hg	4.0 ± 1.4	4.3 ± 1.6	3.9 ± 1.3	3.6 ± 0.2
	Median(Q1,Q3)	3.8(3.0,4.6)	4.3(3.1,4.7)	3.5(2.9,4.7)	3.7(3.4,3.8)
	EOA, cm^2^	2.2 ± 0.3	2.1 ± 0.3	2.2 ± 0.3	2.2 ± 0.2
	Median(Q1,Q3)	2.2(2.0,2.4)	2.1(1.9,2.4)	2.2(2.1,2.4)	2.3(2.0,2.4)
*P*-value for trend^*^	PHT	0.296	0.296	0.296	1.000
	MG	1.000	0.540	0.540	1.000
	EOA	1.000	0.540	1.000	1.000

*MG, mean gradient; EOA, effective orifice area; PHT, pressure half time*.

* *Mann–Kendall test for trend analysis*.

## Discussion

This prospective, multicenter, single-arm clinical trial investigated the clinical safety and hemodynamic outcomes of the Cingular bovine pericardial valve in a patient cohort of 197 patients. The 2-year valve hemodynamics and safety outcomes were good.

For patients between 50 and 65 years who require AVR and who are not contraindicated to anticoagulation, the 2020 ACC/AHA guideline recommends that either a mechanical or bioprosthetic AVR is reasonable after consideration of individual patient factors and informed shared decision-making (8). Thus, an increase in use of bovine pericardial valves in the near future may be a reasonable assumption. However, the long-term durability of bioprosthetic valves is still mainly influenced by SVD. The process of SVD is caused by biologically derived valvular leaflet tissue undergoing calcification over time, leading to stiffening and tearing (16). Many commercially available artificial heart valves undergo constant modification over time, including the well-known Carpentier-Edwards Perimount valve (17, 18). Recently, with the integrity preservation technology, the RESILIA™ tissue leaflets have exhibited excellent midterm outcome with no SVD during a 5-year follow-up period (19).

In addition, the potential factors that may have effects on the rate of calcification are: the extent of patient-prosthesis mismatch (PPM) and the amount of mechanical stress in the valve (20, 21). Apart from the leaflets, the structure of the bioprosthetic valve also influences the stress on the leaflets and the durability of the valve. Thus, the Cingular bovine pericardial valve mainly focuses on the improvement of the stent design. In brief, the novel Cingular valve three-layer stent design intends to keep the stent circular both in stationary and stressed states. The groove between the internal and external polyester rings is provided to fix the alloy wire, ensuring that perfect matching between the stent and the alloy wire can be achieved to avoid malposition (10). Mismatch between the stent and alloy wire may lead to a shift in position of the alloy wire from the stent, potentially causing a decreased stability of the entire valve and uneven stress on the tissue leaflets ultimately resulting in wrinkles and expediting wear of the tissue leaflets. For all the patients completing the 2-year follow-up in our study, no SVD was found in any of the patients. The short-term results were promising; however, it should be recognized that SVD is infrequent in the first few years (20). Thus, long-term durability of this study valve must be validated by a longer follow-up.

The hemodynamic performance of stented tissue valves is clinically important. In particular, this is the case for small size valves. Small EOAs exhibit increased gradients and also limit the possibility of valve-in-valve reintervention in the future. However, in the case of the contemporary stented bioprosthetic valve, the EOAs of 19- and 21-mm sizes remain unsatisfactory. Nishioka et al. (22) reported EOAs of 1.3 ± 0.2, 1.3 ± 0.2, and 1.3 ± 0.2 cm^2^ for the 19-mm Carpentier-Edwards Perimount, Magna, and the Magna Ease valves, respectively, and values of 1.5 ± 0.3 cm^2^, 1.6 ± 0.2 cm^2^, and 1.4 ± 0.3 cm^2^ for the 21-mm valve size, respectively, at least 6 months postoperatively in elderly Japanese patients. The COMMENCE trial reported EOAs and mean gradients of 1.1 ± 0.2 and 1.3 ± 0.3 cm^2^ and 17.6 ± 7.8 and 12.6 ± 4.7 mm Hg, respectively, for 19- and 21-mm sizes at 1 year for the Carpentier-Edwards Perimount Magna Ease with RESILIA™ tissue (23). The EOA of 19-mm Carpentier-Edwards Perimount™ Magna Ease valves with RESILIA™ tissue decreased to 1.0 cm^2^ after 5 years (19).

The abovementioned optimizations in the design of the Cingular bovine pericardial valve offered to not only increase the stability of the valve but also decrease the extent of triangular leaflet opening to make the EOA larger. Stented tissue valve designs are commonly designed with relatively voluminous sewing rings, which tend to decrease the EOA and increase the potential for PPM, particularly in patients with small aortic annuli (24). The sewing ring was, therefore, also redesigned. The outcome of this study reveals excellent 2-year postoperative hemodynamic results for the Cingular bovine pericardial valve, including the smaller sized aortic valves (19 and 21 mm). The incidence of PPM in the aortic valve position was also very low (1.3%) (11).

## Limitation

This trial was set up as a non-randomized, non-comparative, single-arm study. As a consequence, selection bias cannot be excluded. For that matter, randomized controlled trials will be necessary in the future to allow comparison with other valves. The current study was limited to a 2-year outcome period. In order to demonstrate long-term safety and effectiveness, additional follow-up will be required, and it is our intention to follow these patients up for 5 years postoperatively.

## Conclusions

The current clinical investigation with the Cingular bovine pericardial valve revealed good safety and hemodynamic outcomes for surgical aortic and mitral valve replacement at 2 years of follow-up. No SVD events were observed, and excellent hemodynamic performance was seen, even in the case of the smaller sizes of aortic bioprostheses. Still, additional longer-term data will be required to validate the long-term safety and efficacy of this valve.

## Data Availability Statement

The raw data supporting the conclusions of this article will be made available by the authors, without undue reservation.

## Ethics Statement

The studies involving human participants were reviewed and approved by Zhongshan Hospital, Fudan University, West China Hospital of Sichuan University, Wuhan Asia Heart Hospital, The Second Xiangya Hospital of Central South University, and The First Hospital of China Medical University. The patients provided their written informed consent to participate in this study.

## Author Contributions

YG, LT, XZ, TG, LW, TH, and CW contributed to conception and design of the study. JC, YL, and JF organized the database. ML performed the statistical analysis. JC wrote the first draft of the manuscript. JC, YL, and ML wrote sections of the manuscript. All authors contributed to manuscript revision, read, and approved the submitted version.

## Funding

This work was supported by the Shanghai Municipal Commission of Health and Family Planning (Grant No. 201540385), the Shanghai Sailing Program (No. 20YF1405400), the fellowship of China Postdoctoral Science Foundation (No. 2020M671001), and the Youth Research Fund of Zhongshan Hospital, Fudan University (No. 2020ZYYS-003). Shanghai Cingular Biotech Corporation provided the study valve free of charge.

## Conflict of Interest

The authors declare that the research was conducted in the absence of any commercial or financial relationships that could be construed as a potential conflict of interest.

## Publisher's Note

All claims expressed in this article are solely those of the authors and do not necessarily represent those of their affiliated organizations, or those of the publisher, the editors and the reviewers. Any product that may be evaluated in this article, or claim that may be made by its manufacturer, is not guaranteed or endorsed by the publisher.
